# SARS-CoV-2 Testing Behavior in Symptomatic Adults and the Role of Exposure Risk, Susceptibility and Healthcare Access in a U.S. National Cohort (2020–2023)

**DOI:** 10.21203/rs.3.rs-5194738/v1

**Published:** 2025-06-03

**Authors:** Jenna Sanborn, Saba Qasmieh, Kate Penrose, Angela Parcesepe, Yanhan Shen, Rachael Piltch-Loeb, Josefina Nunez, Denis Nash, McKaylee Robertson

**Affiliations:** Institute for Implementation Science in Population Health (ISPH), City University of New York; Institute for Implementation Science in Population Health (ISPH), City University of New York; Institute for Implementation Science in Population Health (ISPH), City University of New York; Department of Maternal and Child Health, Gillings School of Public Health, University of North Carolina; Institute for Implementation Science in Population Health (ISPH), City University of New York; Institute for Implementation Science in Population Health (ISPH), City University of New York; Institute for Implementation Science in Population Health (ISPH), City University of New York; Institute for Implementation Science in Population Health (ISPH), City University of New York; Institute for Implementation Science in Population Health (ISPH), City University of New York

**Keywords:** SARS-CoV-2 testing, COVID-19, diagnostic testing, viral test, antigen test, self-tests, at-home tests, healthcare access, equity

## Abstract

**Background:**

The COVID-19 pandemic highlighted the critical role of diagnostic testing for managing transmission and reducing the risk of serious illness. This study examines SARS-CoV-2 testing behavior patterns, including at-home and laboratory tests, among adults with COVID-like symptoms from 2020–2023. We explore characteristics associated with testing frequency and assess the impact of SARS-CoV-2 exposure risk, susceptibility to COVID-19 complications, and barriers to healthcare access on frequency of testing when ill.

**Methods:**

The CHASING COVID Cohort study comprises a diverse sample of U.S. adults, with data collected quarterly from March 2020 to December 2023. We categorized participants with COVID-like symptoms reported 2+ times (N = 3,929) as ‘frequent testers’ if they tested ≥ 66% of the time when symptomatic, ‘occasional testers’ if they tested > 33% and < 66% of the time, and ‘infrequent testers’ if they tested ≤ 33% of the time. Informed by the Blumenshine Pandemic Disease Framework, we examined the impact of SARS-CoV-2 exposure risk, susceptibility to COVID-19 complications if infected, and barriers to healthcare access on testing frequency using crude and adjusted log-binomial regression models.

**Results:**

Infrequent testers were more likely to be female, Hispanic or Black/non-Hispanic, have an annual household income <$35,000, have fewer years of education, have children in the household, reside in a rural area or southern state. Testing frequency was positively correlated with COVID-19 vaccination, willingness to take antivirals, trust in public health agencies and healthcare providers for COVID-19 vaccine information. Those with more (versus less) exposure risk (aOR 1.14, 95% CI [1.01, 1.26]), COVID-19 susceptibility (aOR 1.17, 95% CI [1.05, 1.30]), no insurance (aOR 1.52, 95% CI [1.37, 1.70]), and no regular healthcare provider (aOR 1.32, 95% CI [1.19, 1.46]) were more likely to test infrequently. Those with more (versus less) exposure risk, susceptibility, and barriers to healthcare were less likely to have purchased SARS-CoV-2 at-home tests and to have requested freely available tests from covidtests.gov.

**Conclusions:**

Initiatives to increase testing uptake should prioritize reaching those with greater risk of SARS-CoV-2 exposure, susceptibility to severe COVID-19, and for those with barriers to healthcare access.

## Background

The COVID-19 pandemic highlighted the critical role of diagnostic testing and screening in managing infectious disease outbreaks. (1,2) Diagnostic testing identifies SARS-CoV-2 infected individuals, aids surveillance, informs public health responses, and helps individuals understand their need for isolation and potentially access to antivirals and medical care.(1) Since March 2020, the US Centers for Disease Control and Prevention (CDC) has consistently recommended SARS-CoV-2 testing for individuals presenting with signs and symptoms of COVID-19 or known exposure to the virus.(3–5) Despite consistency in recommendations for when to seek SARS-CoV-2 testing, the availability of tests fluctuated throughout the pandemic. By mid-2020, both molecular and rapid antigen tests were generally available at public health, clinical and commercial laboratories and at-home tests were approved for emergency use in November 2020.(6–8) By May 2023, more than 50 million diagnostic tests had been made freely available via federal initiatives at clinical, pharmacy and community-based sites. (9) Additionally, more than 750 million free at-home SARS-CoV-2 tests- 4 per household- were distributed to more than 80 million households via covidtest.gov in January 2022, with further rounds of free tests offered in November 2023 and September 2024.(8,10) Research has shown that while federal initiatives improved access to testing, disparities in uptake persisted based on socioeconomic, racial, and regional factors.(10–12)

The Blumenshine pandemic disease framework, adapted from Diederichsen et al., identifies disparities in virus exposure, susceptibility to illness if infected, and access to timely and effective treatment along socioeconomic and racial/ethnic lines as critical factors contributing to unequal rates of illness and death. (13,14) These factors- potential exposure to SARS-CoV-2, susceptibility to COVID-19 complications, and barriers to healthcare- may contribute to disparities in COVID-19 morbidity/mortality by affecting both access to SARS-CoV-2 testing (e.g. via health insurance, ability to purchase at-home tests, or awareness and access to free testing initiatives), and the decision to get tested. Uptake of testing is important since test results can influence behaviors that affect transmission and reduce the risk of serious illness; persons who know they are contagious can more promptly isolate and access care and treatment. Disparities in testing and who is accessing and using tests can also impact the accuracy of public health surveillance.(15,16) Previous research investigating trends in SARS-CoV-2 testing behavior has included cross-sectional and longitudinal studies focused on perceived abilities to access testing(17), testing preferences(18), or disparities in characteristics of at-home testers or those using laboratory tests.(18–23) To our knowledge, no studies have focused on the frequency of testing throughout the pandemic or the impact of contributing factors to pandemic disparities on testing behavior.

In a national community-based cohort, we investigated whether individuals who reported symptoms of COVID-like illness also reported taking a SARS-CoV-2 viral test, and identified sociodemographic characteristics and COVID-19-related perceptions and behaviors associated with the frequency of testing when symptomatic. Guided by the pandemic disease framework, we examined how potential SARS-CoV-2 exposure, susceptibility to COVID-19 complications, and barriers to healthcare access influenced the likelihood of seeking testing when symptomatic. By examining SARS-CoV-2 testing patterns among symptomatic individuals, this study aims to provide insights that can improve testing accessibility and uptake, in order to mitigate future respiratory virus transmission and severe disease outcomes.

## Materials & Methods

### Data Source and Population

The CHASING COVID Cohort study enrolled a geographically and sociodemographically diverse sample of adults (18 years and older) residing in the U.S. or U.S. territories during the emergence of the COVID-19 pandemic.(24) Recruitment and follow-up of the cohort have been described elsewhere.(25) Briefly, participants were recruited and enrolled online between March 28-August 21, 2020, using web-based strategies such as social media advertisements and word-of-mouth referrals. Participants completed online assessments approximately every three months from enrollment through December 2023. This analysis included the participants who completed both the baseline assessment and reported symptoms consistent with the Council of State and Territorial Epidemiologists (CSTE) definition of COVID-like illness in at least two follow-up assessments between July 2020 and December 2023. Sixteen assessments conducted during this period included information on both symptoms and SARS-CoV-2 testing, and were analyzed in this study. Study materials, including assessments, are accessible online.(26) This study was approved by the Institutional Review Board at the City University of New York. Participant consent was obtained at enrollment and at periodic follow-up assessments.

### Sociodemographics

Age, gender, race/ethnicity, educational attainment, household income, employment status, any chronic conditions, children in the household, US state of residence, and ZIP code were collected at enrollment. ZIP code was used to create the geographic-level variable for residential area type, which was assigned based on the NCES Education Demographic and Geographic Estimates locale definitions, using the ZCTA locale file to map ZIP codes to ‘Rural’, ‘Suburban’, ‘Urban’, and ‘Town’. Given the low number of ‘Town’ designations (n=7), ‘Suburban’ and ‘Town’ were collapsed into a single category. Geographic region was determined by the US state of residence collapsed into four geographic regions, ‘Midwest’, ‘Northeast’, ‘South’ and ‘West’, based on U.S. Census Bureau designations.(27)

### COVID-19 perceptions and behaviors

We collected data on COVID-19 vaccination, vaccine concerns, trusted sources of vaccine information, willingness to use SARS-CoV-2 antivirals, at-home SARS-CoV-2 test purchases, and requests for free tests from the federal government. Survey questions can be found in Supplemental Table 4. We determined vaccination status based on reported receipt of both the COVID-19 primary vaccine series, defined as either one dose of a single-dose vaccine (e.g., Janssen) or two doses of a two-dose vaccine (e.g., mRNA vaccines), in addition to at least one additional dose following the primary series by December 2023. To evaluate concerns about COVID-19 vaccines, participants were presented with ten claims related to vaccine safety, efficacy and ill-intent subscales in the October 2022, April 2023, and September 2023 assessments.(28) Participants indicated their level of agreement, disagreement, or uncertainty with each statement. Those who agreed with any false claims or disagreed with any true claims were identified as having vaccine concerns. We dichotomized vaccine concerns as endorsement of concerns in any assessments; otherwise not.

Participants were asked to select whom they trust to provide reliable information regarding the COVID-19 vaccine from a list of sources in the October 2022, April 2023 and September 2023 assessments. Responses were used to construct two binary variables: 1) Trust in federal or intergovernmental public health institutions, the Centers for Disease Control (CDC), World Health Organization (WHO), or Food and Drug Administration (FDA), and 2) Trust in healthcare providers, including a personal physician or other healthcare provider/worker. Those endorsing any of these response options in any of the three assessments were classified as having trust, while those who did not endorse any were classified as having no trust.

In December 2021, when antivirals first became available, we asked whether participants would take an antiviral pill meant to prevent severe COVID-19. We constructed a binary variable indicating participants endorsing the ‘very likely’ response option versus all others. Participants were asked about household purchases of at-home test kit(s) in the December 2021 and March 2022 assessments. Those endorsing having purchased at-home test kits in either assessment were categorized as ‘yes’, and ‘no’ otherwise. Additionally, in the March 2022 assessment, we assessed if participants or their household members requested free COVID tests from covidtest.gov, and created a similar binary indicator.

### Dependent Variable: Infrequent, Occasional and Frequent Testing when Symptomatic

At each assessment, participants were asked about symptoms they experienced since their last assessment. Those reporting symptoms consistent with the August 2020 CSTE clinical criteria for COVID-like illness were classified as having COVID-like illness for that assessment, which required the presence of at least two of the following symptoms: fever (measured or subjective), chills, shivering, body aches, headache, sore throat, loss of taste or smell; or at least one of the following symptoms: cough, shortness of breath, or difficulty breathing.(29) For participants reporting symptoms of COVID-like illness, we tabulated reports of taking a viral test (either PCR or rapid antigen) during the assessment where they reported COVID-like illness. Participants were then categorized as frequent, occasional, or infrequent testers based on the proportion of assessments in which they both reported symptoms and took a viral test, relative to the total number of assessments in which they reported symptoms. We categorized testers based on the frequency with which they tested for COVID-19 when experiencing COVID-like symptoms. We defined *a-priori* cut-offs for infrequent, occasional and frequent testers as testing < 33%, >33% and < 66%, and >=66% of the time, respectively.

### Independent Variables: Potential for SARS-CoV-2 Exposure, Susceptibility to COVID-19 Complications, and Barriers to Healthcare Access

To measure potential for SARS-CoV-2 exposure, we created a summative index consisting of environmental and work-related factors related to household crowding, public transportation use, ability to work from home, dependence on childcare outside of the home, and urban poverty, which Blumenshine et al. highlight as contributing to reduced ability to social distance and increasing potential virus exposure risk.(13) Measurement details are provided in Supplemental Table 4. Each endorsed measure was assigned a value of 1, and these values were summed to create an overall exposure index. This index was then dichotomized at the median (>2), classifying participants into groups with higher versus lower potential SARS-CoV-2 exposure.

As a measure of COVID-susceptibility, we used factors identified by the CDC in March 2020 as increasing the risk for severe COVID-19, if SARS-CoV-2 infected(30). These included age ≥60 years, daily smoking and underlying chronic conditions (chronic obstructive pulmonary disease (COPD), emphysema, chronic bronchitis, angina/coronary heart disease, high blood pressure, history of myocardial infarction, current asthma, type 2 diabetes, kidney disease, immunocompromised condition, or HIV-positive). Respondents endorsing any of these were considered susceptible to COVID-19 complications. A similar index has been used in influenza surveys and our previous work to indicate potential risk for exposure to a respiratory virus and susceptibility to complications, if infected.(30–32)

As markers of barriers to healthcare access, we separately examined two variables: health insurance status and having a regular provider of care. Health insurance status was assessed at each timepoint: we summed the number of assessments where participants reported either not having insurance or not knowing if they had insurance. Participants endorsing these responses in two or more assessments were classified as uninsured. Those who indicated having insurance in at least one assessment were classified as insured. Participants were classified as not having a regular provider if they reported *not* having one or were unsure if they had one in December 2021 or September 2023; baseline data was used if those assessments were missing.

### Confounders

Using a directed acyclic graph framework (Supplemental Figures 1–4), we identified confounders *a-priori* and used the minimum sufficient adjustment set to estimate the total effect of each independent variable on testing frequency. For all models, confounders in the final adjustment sets included age, race/ethnicity, annual household income, employment status, presence of children in the household, and residential area type (urban, suburban, or rural). The susceptibility model also adjusted for having a regular healthcare provider and health insurance status. The health insurance and provider of care models further adjusted for having a chronic condition or being a daily smoker, while the regular provider of care model additionally adjusted for health insurance status.

### Statistical analysis

We conducted bivariate analyses for categorical variables using the adjusted Pearson χ^2^ and Fisher’s exact tests to describe the distribution of participant characteristics across the three testing frequency categories, as well as across exposure, susceptibility and healthcare access groups. Contingency table analyses were performed using Stat Calc 2.2 to assess pairwise differences between groups. We ran crude and adjusted log-binomial regression models for dichotomous outcomes to examine the impact of each independent variable (high potential SARS-CoV-2 exposure, susceptibility to severe COVID-19, or barriers to healthcare access (lacking health insurance and regular healthcare provider) on testing frequency. We compared infrequent testers to frequent/occasional testers and calculated 95% confidence intervals (CIs). Statistical significance was set at p < 0.05. All other analyses were conducted using SAS 9.4 (SAS Institute, Cary, NC, USA).

### Sensitivity Analysis

To explore whether another diagnosis may explain reported symptoms and thus impact SARS-CoV-2 testing behavior, we hypothesized that conditions mimicking COVID-19 (e.g., asthma, COPD, seasonal allergies) would result in disparities in testing frequency, while conditions that do not mimic COVID-19 (e.g., high blood pressure) would not. We conducted bivariate analyses for each chronic illness using the adjusted Pearson χ^2^ to examine the distribution of participant characteristics across three testing frequency categories.

## Results

### Symptoms and Testing Frequency

The analytic sample was comprised of 3,929 participants who reported symptoms consistent with COVID-like illness in at least two out of 16 assessments (median=5 assessments with symptoms, IQR=5). About a third of participants (30%) had more potential SARS-CoV-2 exposure risk (n=1,178), 51% were more susceptible to COVID-19 complications (n=1,989), 24% were uninsured (n=940), and 34% had no regular provider of care (1,325). A quarter (25%) of participants were frequent testers (n=968), 47% were occasional testers (n=1,832), and 29% were infrequent testers (n=1,129) (Table 1). Infrequent testers were least likely to have completed all 16 assessments (68% frequent and 67% occasional vs. 62% infrequent, p=0.0015) and were more likely than frequent testers to report symptoms consistent with COVID-like illness at least 10 times (19% vs. 10%) (Supplemental Table 1).

### Sociodemographics

There were no significant differences in age or essential worker status across testing frequency groups (p> 0.1). However, infrequent testers were more likely than occasional or frequent testers to be female (55% infrequent/ 53% occasional vs. 49% frequent), Hispanic (20% vs. 16% vs. 13%) or Black, non-Hispanic (13% vs. 8% and 8%), have an annual household income <$35,000 (36% vs. 26% vs. 21%), have children living in the household (35%vs.31%vs28%), reside in a rural area (38%vs.30%vs.24%) and reside in the South (36% vs. 32% vs. 26%) and were less likely to be college graduates (52% vs. 65% vs. 74%) (Table 3).

### COVID-19 perceptions and behaviors

Infrequent testers were least likely to have received their primary vaccination series plus at least one additional dose (70% vs. 83% vs. 93%), to be very willing to take an antiviral (53% vs. 60% and 62%), to purchase at-home tests (18% vs. 36% vs. 50%), to order free tests on covidtests.gov (62% vs. 80% vs. 87%), and to trust public health institutions (82% vs. 89% vs. 95%) or healthcare providers (77% vs. 86% and 89%) for COVID-19 vaccine information. They were most likely to have vaccine concerns (62% vs. 46% vs. 34%) (Table 3).

### SARS-CoV-2 Exposure, COVID-19 Susceptibility, and Healthcare Access

Participants with more potential SARS-CoV-2 exposure risk had 14% greater likelihood to be an infrequent tester (aRR 1.14, 95% CI 1.01, 1.26) compared to those with less exposure risk. Participants with more susceptibility to COVID-19 complications had 17% greater likelihood of being an infrequent tester (aRR 1.17, 95% CI 1.05, 1.30) compared to those not classified as susceptible. Barriers to healthcare as measured by lack of health insurance increased the risk of being an infrequent tester by 52% (aRR 1.52, 95% CI 1.37, 1.70), while no regular healthcare provider increased this risk by 32% (aRR 1.32, 95% CI 1.19, 1.46) compared to those that have insurance and a regular provider, respectively (Table 2).

### Test Purchasing and Requests for Free SARS-CoV-2 Tests

Between December 2021 and March 2022 (Omicron era), individuals with more (versus less) potential exposure risk were less likely to purchase at-home SARS-CoV-2 tests (32% vs. 35%), as were those with more susceptibility (31% vs. 38%), no health insurance (64% vs. 80%), and no regular healthcare provider (68% vs. 80%). Similarly, those with more exposure risk were less likely to have requested free at-home tests from covidtests.gov (71% vs. 78%), as were those with more susceptibility (75% vs. 78%), no health insurance (64% vs. 80%), and no regular healthcare provider (68% vs. 80%) (Table 1). Both test purchasers and requesters were more likely to be White/non-Hispanic, college graduates, have annual household income ≥$100,000, and urban residents (Supplemental Table 2).

### Sensitivity Analysis

Infrequent testers were more likely to have high blood pressure (26%/23% vs. 18%), type 2 diabetes (9%/7% vs. 4%), and COPD, emphysema, or chronic bronchitis (5% vs. 3% vs. 2%), compared to occasional and frequent testers. There were no significant differences in the prevalence of asthma or seasonal allergies between testing frequency groups (p>0.1) (Supplemental Table 3).

## Discussion

We examined the frequency of SARS-CoV-2 testing among individuals experiencing COVID-like symptoms from 2020 to 2023, which has important implications for SARS-CoV-2 transmission, surveillance and long-term health outcomes. (2, 15, 16) We found that those who would benefit most from SARS-CoV-2 testing–individuals with more risk of SARS-CoV-2 exposure and more susceptibility to COVID-19 complications–were the least likely to test when symptomatic. Infrequent testers were also more likely to report symptoms of COVID-like illness, indicating that being sick more often may lead to testing fatigue. Failing to test can contribute to virus transmission, as those who are unaware of their infection are less likely to isolate, while infrequent testing among those more susceptible to COVID-19 complications limits timely access to treatment, resulting in missed opportunities for early intervention with COVID-19 treatments which often require a prescription within a week of symptoms.(30,33) Previous research has shown that 82% of antiviral-eligible adults do not consider themselves high-risk for COVID-19 complications, which may contribute to reduced likelihood to seek testing.(34) Public health efforts should focus on educating at-risk populations about the associated risks of COVID-19 complications and emphasize the importance of getting tested when ill, including if symptoms are mild. Furthermore, those with the greatest barriers to healthcare access were also the least likely to test. This may be due to concerns about cost, or may be due to the fatalistic belief that testing offers little benefit if treatment is inaccessible; this aligns with the Theory of Planned Behavior, where attitudes about testing’s usefulness and perceived control over actions once tested shape intentions to seek testing.(35, 36) However, delays in testing may lead to seeking care only when their condition has worsened, reducing the effectiveness of treatments and increasing the risk of complications.

Consistent with previous studies, we identified socio-economic status as an important predictor of likelihood to test for SARS-CoV-2 when ill;(17, 37) infrequent testers were more likely to be female, be Hispanic or Black/non-Hispanic, have an annual household income <$35,000, have fewer years of education, have children in the household, and to reside in a rural area or a southern state. Infrequent testers were also more likely to have concerns about the vaccine, and less willing to take antivirals or to trust public health institutions and healthcare providers for COVID-19 vaccine information. Thus, testing behavior was also found to be linked to other COVID-19 beliefs and behaviors, substantiating the importance of trust-building efforts in bolstering adherence to public health messaging.(38, 39)

Our findings also reveal that individuals with lower socio-economic status, higher exposure risk, greater susceptibility to COVID-19 complications, and more barriers to healthcare access were also less likely to purchase at-home tests or request free tests from covidtests.gov, highlighting persistent disparities in testing access. While the federal testing program increased overall access–35% purchased at-home tests and 76% ordered free tests from covidtests.gov–these disparities remained, suggesting that the initiative achieved equality by offering tests widely, but not equity in reaching those most in need. To address disparities in testing, future public health strategies should focus on targeted outreach and support to ensure equitable access. Ensuring nationwide Medicaid coverage for uninsured people, especially during a pandemic, may help reduce disparities. SARS-CoV-2 testing initiatives may consider offering no or very low cost at-home tests ($1-$2) or allow for those without insurance to receive free tests at any doctors’ office, hospital, or urgent care site, rather than rely on free testing locations, alleviating logistical and financial barriers for those with a preference for provider-based testing.(17) Indeed, access to and utilization of home tests has been shown to be higher among those with greater socio-economic status.(19) A nationally-representative study showed lower uptake of at-home tests, but greater uptake of provider-based tests among non-Hispanic Black adults;(31) Upfront costs associated with at-home tests may impose financial barriers that clinic-based testing does not.

A strength of this study is its use of longitudinal data, enabling insight into testing patterns throughout the course of the pandemic. However, several limitations were considered. First, data is self-reported and subject to recall bias. Further, the study population, while geographically and socioeconomically diverse, is not representative of the U.S. population, limiting generalizability. Moreover, our methods assumed that symptoms and testing reported in a given assessment occurred simultaneously, but this was not explicitly assessed. Additionally, we were unable to determine if participants were subject to testing requirements or universal screening testing programs, as required by many schools and workplaces, potentially affecting testing behavior.(40–42) We were also limited in our ability to detect severity of symptoms or if they were new or ongoing, which could have influenced testing behavior. Finally, our sensitivity analysis found that COPD may have contributed to reported symptoms (e.g. shortness of breath), however, the low number of respondents in this category (n = 36) suggests this factor alone is unlikely to drive overall associations.

## Conclusion

Our study highlights that individuals with the greatest risk of SARS-CoV-2 exposure, susceptibility to COVID-19 complications, and barriers to healthcare access were the least likely to test when ill. This has significant public health implications, as failure to test limits early intervention opportunities and contributes to ongoing transmission. Public health strategies should prioritize at-risk populations and ensure that both testing initiatives and access to treatment are accessible. Such efforts will be essential as new variants and public health challenges emerge.

## Data Availability

Study data from the CHASING COVID Cohort is publicly available on Zenodo (DOI: 10.5281/zenodo.6127734). Some data elements and surveys from the CHASING COVID cohort study are not made publicly available due to funder requirements, but this information can be made available to researchers with approved projects.

## Abbreviations

(CDC): Centers for Disease Control and Prevention
(COPD): Chronic Obstructive Pulmonary Disease
(Cls): Confidence Intervals
(CSTE): Council of State and Territorial Epidemiologists
(FDA): Food and Drug Administration
(WHO): World Health Organization
